# The Transcriptome of *Bathymodiolus azoricus* Gill Reveals Expression of Genes from Endosymbionts and Free-Living Deep-Sea Bacteria

**DOI:** 10.3390/md10081765

**Published:** 2012-08-20

**Authors:** Conceição Egas, Miguel Pinheiro, Paula Gomes, Cristina Barroso, Raul Bettencourt

**Affiliations:** 1 Biocant—Biotechnology Innovation Centre, 3060-197 Cantanhede, Portugal; Email: monsanto@biocant.pt (M.P.); paula.gomes@biocant.pt (P.G.); cristina.barroso@biocant.pt (C.B.); 2 IMAR—Institute of Marine Research, University of Azores, 9901-861 Horta, Portugal; Email: raul@uac.pt

**Keywords:** *Bathymodiolus azoricus*, mussel, Lucky Strike, Mid Atlantic Ridge, deep-sea, thiotrophic endosymbiont, methanotrophic endosymbiont, *Sulfurovum*

## Abstract

Deep-sea environments are largely unexplored habitats where a surprising number of species may be found in large communities, thriving regardless of the darkness, extreme cold, and high pressure. Their unique geochemical features result in reducing environments rich in methane and sulfides, sustaining complex chemosynthetic ecosystems that represent one of the most surprising findings in oceans in the last 40 years. The deep-sea Lucky Strike hydrothermal vent field, located in the Mid Atlantic Ridge, is home to large vent mussel communities where *Bathymodiolus azoricus* represents the dominant faunal biomass, owing its survival to symbiotic associations with methylotrophic or methanotrophic and thiotrophic bacteria. The recent transcriptome sequencing and analysis of gill tissues from *B. azoricus* revealed a number of genes of bacterial origin, hereby analyzed to provide a functional insight into the gill microbial community. The transcripts supported a metabolically active microbiome and a variety of mechanisms and pathways, evidencing also the sulfur and methane metabolisms. Taxonomic affiliation of transcripts and 16S rRNA community profiling revealed a microbial community dominated by thiotrophic and methanotrophic endosymbionts of *B. azoricus* and the presence of a *Sulfurovum*-like epsilonbacterium.

## 1. Introduction

Life in extreme marine environments is highly diverse, as illustrated by deep-sea hydrothermal vents. These structures are located at the mid ocean ridges or subduction zones, thus providing particular environmental conditions, such as high pressure, steep temperature gradients, and potentially toxic concentrations of sulfur, methane, heavy metals and radionuclides. Along the Mid Atlantic Ridge (MAR), several hydrothermal vent fields have been formed, among which the Lucky Strike field, southwest of the Azores Islands, lies at a depth 1700 m. This hydrothermal vent system displays physicochemical properties and water effluxes distinct from other MAR fields, due to a higher sulfur-to-methane ratio, where hydrogen sulfide can reach up to 6 times the concentration of methane [1]. Additionally, the concentration of chemicals such as iron, manganese, copper, zinc, cadmium or lead is usually high, at Lucky Strike, showing intermediate values between the shallower hydrothermal vent Menez Gwen, and the deeper Rainbow hydrothermal vent, located at depths of 850 m and 2300 m, respectively [1].

A complex living community of invertebrates and microbes thrives at deep-sea hydrothermal vents, regardless of the extreme conditions [2]. At the base of the food web, microbes are adapted to the geochemical conditions and dominated by chemoautotrophic bacteria and archaea that oxidize reduced chemicals to fix carbon [3]. Much of the survival success of the invertebrates thriving in these environments relies on the establishment of symbiotic associations with chemoautotrophic bacteria, from which they receive most of their nutrition, whereas the bacteria likely benefit from a protected and stable physical and chemical environment, favorable for carbon fixation [4]. Such symbioses are commonly found among various invertebrate phyla, particularly in bivalve mollusks inhabiting hydrothermal vents, cold seeps, and other suboxic sediments [5], and are structurally supported by specialized organs or tissues harboring methanotrophic, thiotrophic or both bacteria. Enlarged gill tissues containing high densities of endosymbiont bacteria, reduced feeding systems and rudimentary digestive organs constitute the distinct traits of vent bivalves. The mussel *Bathymodiolus azoricus* is the dominant animal at Mid-Atlantic Ridge deep-sea hydrothermal vents, owing its high biomass to the functional dependence on its symbiotic association with both sulfur-oxidizing and methanotrophic bacteria [2,3,6,7]. Such a dual symbiosis is hosted in the bacteriocytes, and may provide the bulk of the host’s nutritional carbon requirement [6,8]. Additional energy sources may be obtained by conventional filter feeding through the ingestion of particulate organic matter, as well as absorption and incorporation of free amino acids [9]. Such mixotrophy provides substantial nutritional advantage to the mussel, not only allowing it to obtain energy from both sulfide and methane at the vent sites, but also from particulate organic matter [10].

The study of biology and genetics of endosymbionts and their relationship with their host is challenging, as these organisms are difficult to grow as free living bacteria in culture media. Nevertheless, the development of new sequencing technologies is fuelling this field of research, and recent high-throughput sequencing approaches applied to genome sequencing, metagenomics and metatranscriptomics now provide a global perspective on taxonomic and functional profiling of microbial communities expectedly under the influence of changing environmental conditions in which they naturally exist.

The metagenomics approach, where genes are sequenced from the genome pool of a microbial community, has been used to characterize deep-sea environments, such as deep-sea communities from Station ALOHA (A Long-term Oligotrophic Habitat Assessment) in the Pacific Ocean [11] or the Mediterranean Sea [12]. While metagenomics is regarded as a static view of the community, the metatranscriptomic approach, in which the genes sequenced are those being expressed, provide the dynamic view of a microbial community in a certain condition or time frame. Such an approach has been used to study the microbial community of ocean surface waters [13] or coastal waters [14]. These omics approaches have also been applied to the study of endosymbiont enzymatic pathways such as the sulfur oxidation pathways in endosymbionts of deep-sea vent clams [15,16] and more recently to the study of *B. azoricus* endosymbionts and their relationship with the host [1].

Using the high throughput 454 pyrosequencing technology, we sequenced the transcriptome of gill tissues from *Bathymodiolus azoricus* to study the biological processes underlying physiological adaptations to hydrothermal vent environments [17]. Sequencing of the gill normalized cDNA library generated 778,996 reads, which assembled into 75,407 contigs, encoding for 39,425 amino acid sequences. We then compiled the transcripts’ nucleotide sequences, encoded proteins and corresponding functional annotation in a dedicated database, the DeepSeaVent. The analysis of this database revealed protein match hits to bacterial phylotypes, supporting evidence for the presence of bacteria in gill tissues of *B. azoricus*, and representing thus a potential bacterial fingerprint, most likely of chemoautotrophic nature, in the deep-sea hydrothermal vent mussels [17].

Here, we report the functional analysis of the transcripts of bacterial origin using MG-RAST-based annotation and reveal the dominant features in gene representation and genes involved in key cellular metabolisms. We additionally describe the diversity and structure of the microbial community through MG-RAST-derived gene affiliation and pyrosequencing of the V6 hypervariable bacterial ribosomal region. 

## 2. Results and Discussion

### 2.1. Overall Patterns of Bacterial Gene Expression in Mussel Gills

Search analysis of DeepSeaVent, the *B. azoricus* gill tissue transcriptome database, revealed the presence of genes matching bacteria phylotypes among the mussel gill transcripts [17]. To understand if these transcripts could represent a bacterial fingerprint in the mussel gill tissues, we searched for transcripts with a BLASTx hit matching the superkingdom Bacteria, and retrieved 3522 contigs [17] (Table 1). We then re-annotated these transcripts using the Meta Genome Rapid Annotation using Subsystem Technology (MG-RAST) [18], a system based on the SEED framework for comparative microbial genomics. After internal quality control and duplicate removal, MG-RAST assigned 1994 transcripts (61.9% of features) to at least one of the M5NR protein databases (GO, IMG, KEGG, NCBI (RefSeq & GenBank), SEED, UniProt, eggNOG and PATRIC), and 90% of these transcripts (1801 transcripts) to functional categories (Table 1). 

**Table 1:** Summary of MG-RAST annotation of the bacterial transcripts found in the *B. azoricus* gill transcriptome.

Description	# Hits
# Transcripts submitted to MG-RAST	3522
Total sequence size submitted (bp)	2,061,462
Sequence length range (bp)	100–3199
Average transcripts length (bp)	622.99
# Transcripts after QC	3099
# Predicted Protein Features	3223
# Identified Protein Features	1994
# Identified Functional Categories	1801
# Transcripts annotated in SEED	918
# Transcripts annotated in KEGG	570
# Transcripts annotated in COG	416

**Figure 1:**
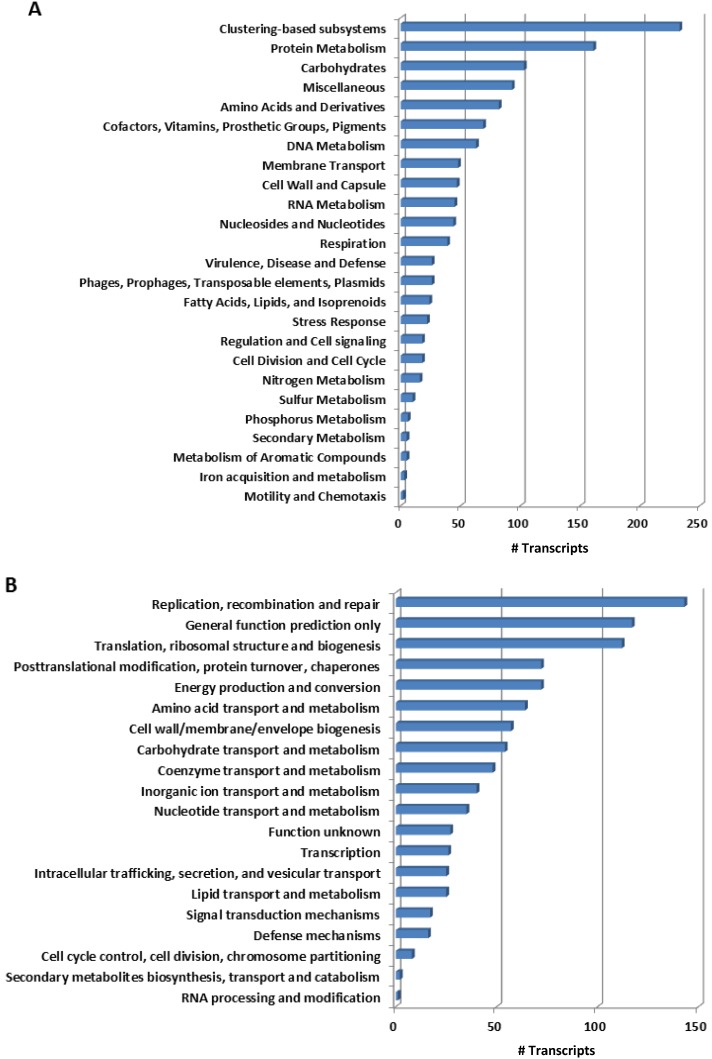
Functional fingerprinting of the bacterial transcripts from mussel gill according to SEED subsystems categories (**A**) and COG (**B**). Annotation parameters were set at a maximum *e*-value of 10^−5^, a minimum of 50% identity cutoff and a minimum alignment cutoff of 50.

We classified the transcripts of *B. azoricus* gill tissues microbial community according to SEED subsystems and to the Cluster of Orthologous Groups (COG) (Table 1) to deduce the functional profile of this community. The bacterial transcripts distributed across 25 subsystems in SEED (Figure 1A, Table S1), where protein metabolism (19% of transcripts), carbohydrates (8.5%) and amino acid and derivatives (6.8%) were the most represented categories. Within these categories, most of the transcripts were involved in the synthesis of amino acids and in translation, whereas most of the transcripts, among carbohydrates, participated in energy production, such as in glycolysis and pyruvate metabolism. Furthermore, we observed a high incidence of transcripts for genes involved in folate synthesis, and for tRNA modification: additional biosynthetic processes. Hence, biosynthesis appears to be the leading function in the mussel bacterial community sample under study, reflecting a metabolically active community. In the lowest represented subsystems, we detected a few transcripts in subsystems that are dispensable for an intracellular lifestyle, such as secondary metabolism (4 transcripts), with transcripts involved in the synthesis of tryptophan or pyridine, and motility or chemotaxis (7 transcripts), where we detected transcripts in the regulation of flagellar motility (Figure 1A, Table S1). 

Genes in these major subsystems are shared by most organisms [19], and, therefore, these categories may not be informative for the identification of transcripts of specialized biochemical pathways [20] particular of this community. Hence, we looked for transcripts in other subsystems such as virulence, disease and defense, or as stress response (Figure 1A, Table S1). We observed incidence of transcripts coding for antibiotic resistance for beta-lactams as well as a transcript for β-lactamase. Additionally, we detected transcripts evidencing metal detoxication from the periplasm to the cell exterior, and the presence of components of type 4 secretion system, involved in conjugative transfer functions and harboring genes involved in biodegradation [21].

Among genes distributed in 17 out of the 23 COG categories, replication, recombination and repair was the most represented group, with 15% of the identified genes (Figure 1B, Table S2). Two thirds of these genes assigned in the COG category of transposases and inactive derivatives, more specifically in COG2801, in COG3328 and COG2826. These results corroborate the findings of other deep-sea studies, where an increase in mobile elements was observed when compared to surface waters, suggesting transposases as a mechanism to introduce phenotypic variability in slow growth rate environments [4,5,20,21]. Nevertheless, we cannot infer on the origin of these genes from the *B. azoricus* endosymbionts or a planktonic community caught in gill tissues at the moment of the animal’s collection from the deep-sea. 

*B. azoricus* thiotrophic endosymbiont is thought to be transmitted through horizontal transfer from the environment [7] indicating that this bacterium may have free and intracellular life forms. Therefore, its genome may not have suffered the loss of non-essential genes and mobile elements as would be expected from a vertically transmitted endosymbiont. The lack of transposases in the genomes of the thiotrophic endosymbionts of the giant hydrothermal vesicomyid clam *Calyptogena* from the Pacific Ocean [16,22] is such an example. Recent studies show an increase in mobile elements from obligate symbionts to facultative symbionts [23,24], and even obligate symbionts that switch between hosts have a higher mobile DNA gene density than the obligate intracellular bacteria that are vertically transmitted [24]. The genome sequencing of the thiotrophic and methanotrophic endosymbionts of *B. azoricus* will certainly help elucidate the presence of mobile elements and their role as a potential mechanism to maintain genetic diversity.

### 2.2. Functional Key Metabolisms in Mussel Gill Bacterial Community

Adding to the functional metabolism overview of the mussel gill bacterial community, we searched for evidence of specific pathways, such as carbon fixation, nitrogen metabolism and the oxidation of sulfur and methane. Considering the physicochemical conditions of the Lucky Strike vent field, markedly with high sulfur and methane concentrations and the predictable presence of thiotrophic and methanotrophic endosymbionts in *B. azoricus* bacteriocytes, we anticipated the detection of transcripts involved in sulfur and the methane oxidation. Indeed, from the gill transcriptome, we verified genes coding for the Sox pathway of sulfur oxidation (Table 2), namely soxB, a putative thiol esterase mediating the hydrolytic release of sulfate from sulfur-bound SoxY, also detected among the transcripts, and soxD, a sulfur dehydrogenase [16]. SoxD is part of a heterotetrameric complex along with soxC [25] and is only active as soxCD. However, we did not detect the counterpart soxC within the transcripts; this may be a consequence of the limited group of transcripts studied. The presence of SoxD is an interesting finding; this gene is absent from the genomes of several green and purple sulfur bacteria and the endosymbionts of deep sea clams and tubeworms [15,26,27], but has been found in the genomes of *Paracoccus pantotrophus* and *Starkeya novella*, two alphaproteobacteria used as models to study the sulfur oxidation pathways [28]. Additionally, transcripts coding sulfite reductase dissimilatory-type beta subunit, dsrB [29], the reversely operating sirohaem dissimilatory sulfite reductase (rDSR) [15,30] were detected, yet the presence of a single gene may not implicate an active pathway for sulfur oxidation to sulfite in the microbial community (Table 2).

The other relevant metabolic pathway expected in this microbial community is methane oxidation. We observed transcripts encoding soluble methane monooxygenase (mmo), subunit C, along with transcripts coding for particulate methane monooxygenase (pmmo). Since this enzyme catalyzes the oxidation of methane to methanol, in free living and endosymbiont methanotrophs [31], it is difficult to deduce the origin of transcripts.

**Table 2:** Transcripts of *B. azoricus* gill bacterial community involved in key metabolisms.

Metabolism/Pathway	Function	Hits #	*e*-Value	Identity (%)	Taxonomic Affiliation
CO_2_ fixation	Rubisco activation protein CbbQ	2	1e^−23^	83.00	*Methylococcus capsulatus*
	Ribose 5-phosphate isomerase A (EC 5.3.1.6)	1	1e^−50^	70.07	*Nitrosomonas*
	Transketolase (EC 2.2.1.1)	4	1e^−59^	74.38	*Methylococcus flagelatus*
	Transketolase, *N*-terminal section (EC 2.2.1.1)	1	1e−^16^	61.43	
Methane oxidation	Methane monooxygenase B-subunit (EC 1.14.13.25)	1	1e^−34^	75.58	*Methylococcus capsulatus*
	Particulate methane monooxygenase C-subunit (EC 1.14.13.25)	1	1e^−44^	85.01	*Methylococcus capsulatus*
Denitrification	Nitrous-oxide reductase (EC 1.7.99.6)	1	1e^−15^	65.52	
Nitrate and nitrite ammonification	Nitrite reductase (NAD(P)H) large subunit (EC 1.7.1.4)	1	1e^−44^	66.93	*Burkholderia*
	Respiratory nitrate reductase alpha chain (EC 1.7.99.4)	1	1e^−46^	78.90	*Hallela*
	Respiratory nitrate reductase beta chain (EC 1.7.99.4)	3	1e^−56^	74.63	*Chromobacterium*
Respiratory dehydrogenases	Methanol dehydrogenase large subunit protein (EC 1.1.99.8)	1	1e^−55^	74.80	*Rhodopseudomonas*
Sulfate reduction-associated complexesSulfur oxidation	Sulfite reductase beta subunit (EC 1.8.99.1)	1	1e^−42^	91.46	*Calyptogena* endosymbionts
	Sulfite dehydrogenase cytochrome subunit SoxD	3	1e^−14^	63.04	Manganese-oxidizing bacterium (strain SI85-9A1)
	Sulfite oxidase	1	1e^−26^	65.38	*Calyptogena* endosymbionts
	Sulfur oxidation protein SoxB	1	1e^−27^	57.73	*Thiobacillus denitrificans*
	Sulfur oxidation protein SoxY	2	1e^−15^	76.16	*Calyptogena* endosymbionts

Transcript gene function was assigned in MG-RAST, with the following parameters: maximum *e*-value of 10^−5^, minimum of 50% identity cutoff and minimum alignment cutoff of 50. Identity (%) refers to amino acid residues that are identical in the hit and the query.

In the analysis of the microbial metatranscriptome, we would also expect to find transcripts involved in carbon fixation, however, RuBisCo, the key enzyme, was not found. Instead, we detected a transcript coding for the RuBisCo activation protein CbbQ, involved in CO_2_ uptake, and two transcripts coding for transketolase and ribose-5-phosphate isomerase A, both involved in the Calvin-Benson-Bassham cycle of autotrophic carbon metabolism [32]. Furthermore, we did not observe any of the key enzymes involved in the nitrification process, such as ammonia monooxygenase (amoA), or other evidence for the presence of ammonia or anammox, in agreement with the studies of Byrne and collaborators [33], that found anammox activity in Lost City and Rainbow, two other hydrothermal vents at MAR. However, we found coding transcripts for enzymes of the denitrification pathway, nitrate and nitrite reductase, and nitrous oxide reductase, suggesting that oxidized nitrogen species can be assimilated to produce nitrogen in this microbial community. A similar enrichment for denitrification enzymes and absence of the nitrification pathway was observed in the Mothra hydrothermal vent field at the Juan de Fuca Ridge [34], suggesting that nitrogen pathways may not be universal but specific for particular vent communities. 

Recent work by Peterson and collaborators [35] suggest the use of hydrogen to power primary production, through hydrogen oxidation. This research group identified a NiFe hydrogenase as a key enzyme in this process. However, in this limited dataset we did not detect any transcript matching this enzyme. A direct search of transcript data with the *hupL* nucleotide sequence did not recover any match. Moreover, specific primers targeting the *hupL* gene could not generate a PCR amplicon using Lucky Strike and Menez Gwen vent mussels cDNA [36]. 

### 2.3. Taxonomic Affiliation of Bacterial Genes

**Figure 2:**
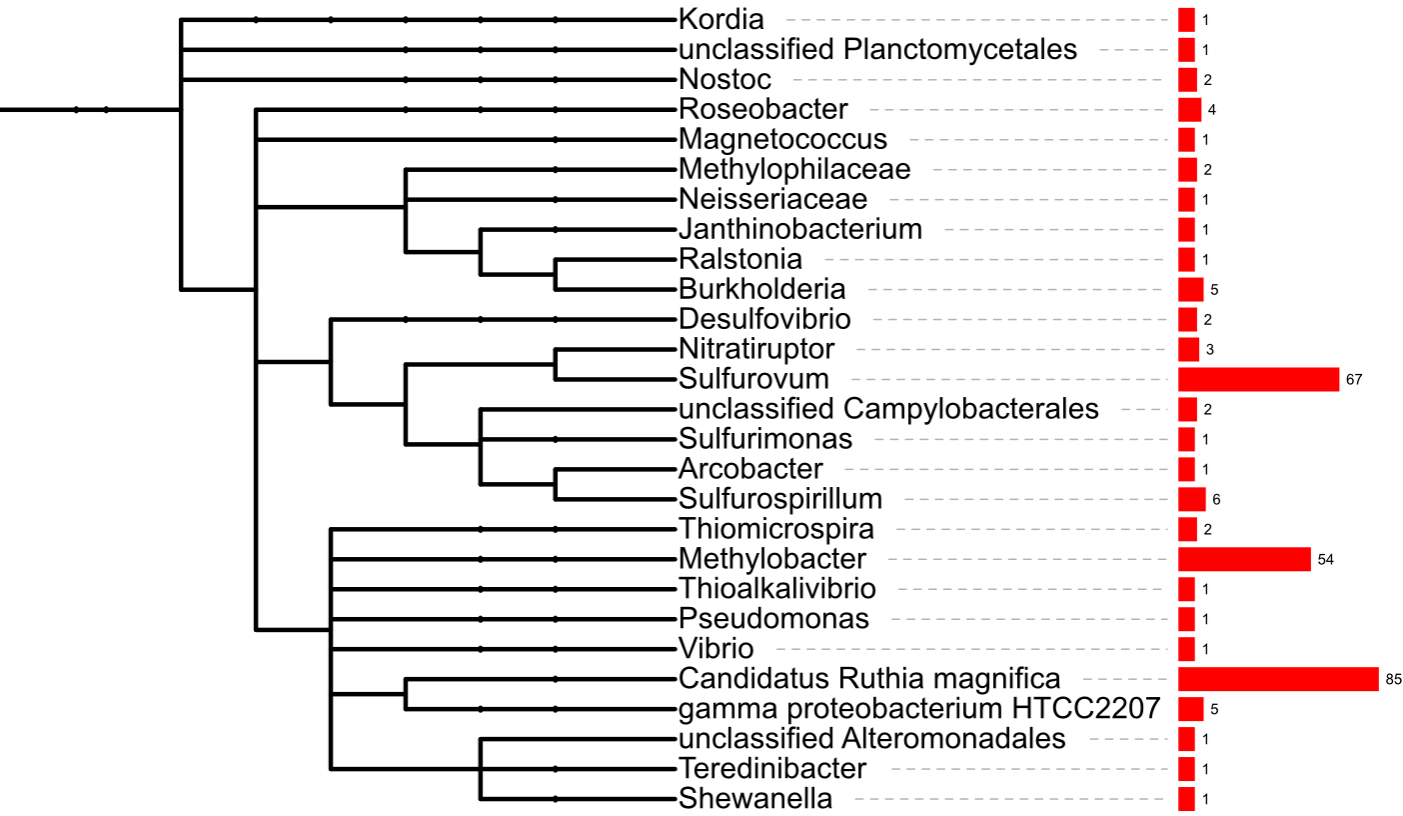
Bacterial fingerprinting of the *B. azoricus* gill. Affiliation of the bacterial transcripts was analyzed with lowest common ancestor algorithm in MG-RAST. The tree was built in ITOL (Interactive Tree of Life [45]) using the NCBI taxon ID of binned microorganisms and corresponding hit abundance.

In addition to assigning the function of genes, the MG-RAST annotation pipeline also identified the organism into which the transcript had the closest BLAST hit, based on genome information available for known organisms in the databases. This process of taxonomic affiliation has been used in metagenome [12,37] and metatranscriptome [19,38] studies to infer on the organisms present in bacterial communities, thus providing an initial community taxonomical analysis. In this study, we used a similar approach to gain an insight into the taxonomic diversity of the mussel gill tissues’ microbial community. Hence, we used the lowest common ancestor method hosted at MG-RAST, after binning transcripts to the M5 non-redundant protein database (M5NR). According to the results obtained, the bacterial transcripts binned almost exclusively to Proteobacteria (99.5% of transcripts), with residual hits in Cyanobacteria (two transcripts), Bacteroidetes (1) and Plantomycetes (1). Within Proteobacteria, 239 transcripts could not bin further to lower taxa, 606 transcripts affiliated in Gammaproteobacteria, whereas remaining genes distributed over Epsilonproteobacteria (127), Betaproteobacteria (10), Alphaproteobacteria (4) and Deltaproteobacteria (2). Within Gammaproteobacteria, the most represented bacterial group was unclassified gammaproteobacteria (39%), probably reflecting yet to be described bacteria, followed by close relatives of the vent clam sulfur oxidizing, Candidatus *Ruthia magnifica* (34%), the endosymbiont of *Calyptogena magnifica* [39] (Figure 2). The second most prevalent binned bacterial group was Epsilonproteobacteria, where *ca*. 50% of the gene hits affiliated in the free-living sulfur-oxidizer *Sulfurovum* [3,40] (Figure 2). The third most represented gene group binned to type I methanotroph *Methylobacter* (21.7%), bacteria able to metabolize methane as the only source of carbon and energy [41] (Figure 2). We additionally detected a lower number of gene hits affiliated in *Sulfurospirillum*, a sulfur reducing bacterium [42]; in gammaproteobacterium HTCC2207 of the SAR92 clade, an oligotrophic bacteria found in sea surface waters [43] and in *Roseobacter*, one of the most abundant marine bacteria, found in sea water and marine sediments [44] (Figure 2).

The taxonomic diversity of the gill tissues bacterial community pinpointed the predominance of hits in the thiotrophic endosymbiont of *Calyptogena magnifica* [27], the high number of transcripts affiliating in *Sulfurovum*, a free living sulfur oxidizer, and in *Methylobacter*, a free living methane oxidizer. However, these results have to be analyzed carefully since bacterial identification heavily relies on the relative representation of closely-related organisms in the reference databases and currently the genome sequences of the thiotrophic and methanotrophic endosymbiont of *B. azoricus* have not been published.

### 2.4. 16S rRNA Analysis of the Microbial Community

To gather further evidence of microbial community diversity in the vent mussel gill, we performed massive parallel pyrosequencing of the bacterial 16S ribosomal RNA. We produced cDNA from the same total RNA sample used for sequencing of the mussel gill’s transcriptome, using random primers in the reverse transcription reaction, as the method to enrich for ribosomal RNA, amplified the V6 hypervariable region with three different primer pairs to maximize the diversity coverage and pyrosequenced the amplicons in a Genome Sequencer FLX. Analysis of high quality sequencing reads resulted in 31 OTUs (Operational Taxonomic Units), assigned into taxonomy by comparison to the Ribosomal Database Project (RDP) [46]. The resulting bacterial identification revealed that more than half of the OTUs had no corresponding matches in databases (Table 3), most likely due to yet non-described deep-sea microbes, supporting evidence that these environments are largely unexplored and may harbor a reservoir of unknown organisms and gene functions. On the other hand, Proteobacteria was the predominant phyla in the bacterial community, with OTUs identified as uncultured gammaproteobacteria, as *Psychromonas*, the piezophilic bacterium adapted to growth at low temperatures and most probably suited for the Lucky Strike’s environmental conditions [47] and the two distinct endosymbionts of *B. azoricus*, the thiotrophic and the methanotrophic endosymbionts (Table 3). Additionally, we found an OTU corresponding to the free living epsilonbacterium *Sulfurovum* [48]. We also observed OTUs corresponding to uncultured *Spirochaetes* that were closely related to those found in the crystalline style in the digestive tract of marine bivalves [49].

**Table 3:** Taxonomic profile of *B. azoricus* gill tissues’ microbial community. The V6 hypervariable region of 16S rRNA was amplified from cDNA and pyrosequenced in a 454 next generation sequencing platform.

Kingdom or Phylum	Class	Genus/Description	OTU	Sequences
Bacteria		Uncultured bacterium	3	13
Proteobacteria	Gammaproteobacteria	Thiotrophic endosymbiont of *B. azoricus*	1	4180
Proteobacteria		Methanotrophic endosymbiont of *B. azoricus*	1	1030
Proteobacteria		*Psychromonas*	1	2
Proteobacteria		Unculturedgammaproteobacterium	4	161
Proteobacteria	Epsilonproteobacteria	*Sulfurovum*	1	1
Spirochaetes		Uncultured Spirochaetes	2	2
Unidentified			18	224

**Figure 3:**
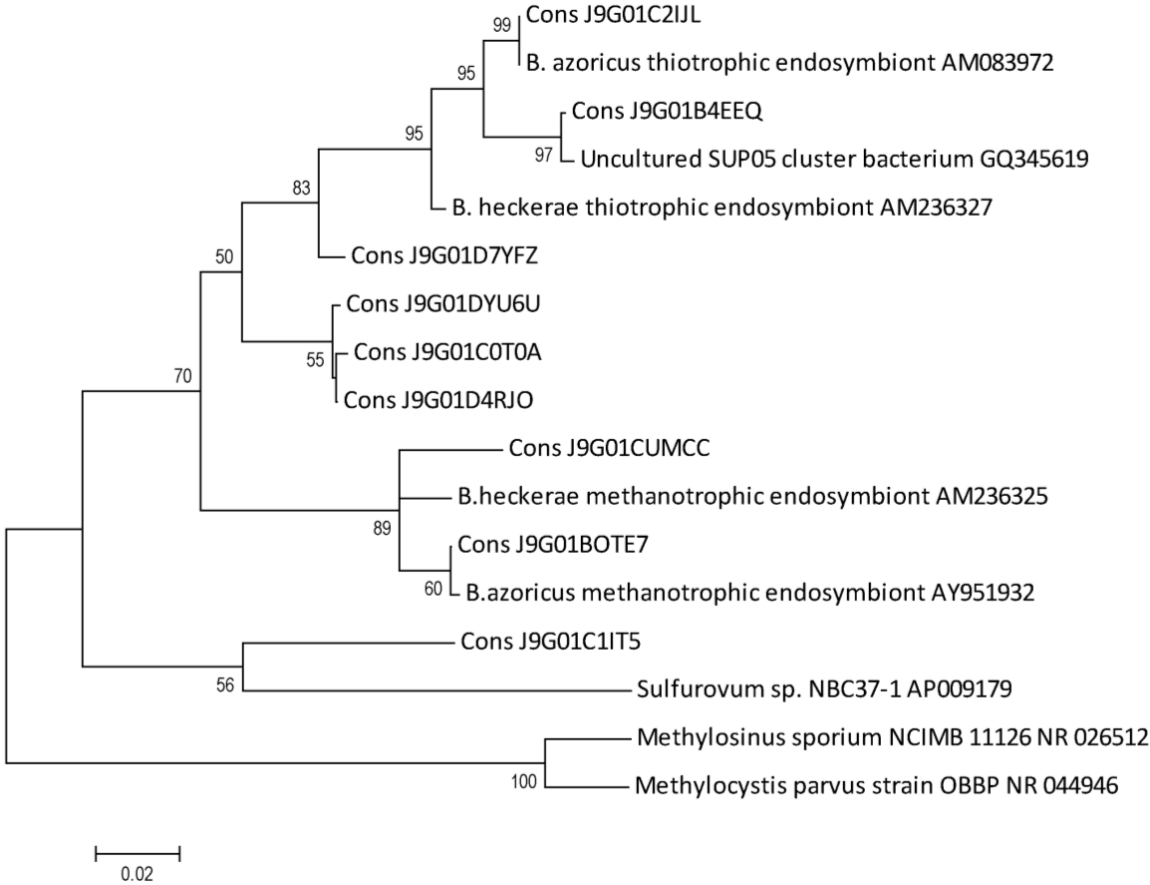
Phylogenetic analysis of V6 rRNA pyrosequencing. Phylogram shows the genetic relationship between the OTUs (Cons X) obtained through pyrosequencing and 16S RNA of closely related strains, retrieved from RDP. The tree was built by the Maximum Likelihood method based on the Jukes and Cantor model, and evaluated by 1000 bootstrap replicates. Only values over 50% are shown. *Methylosinus sporium* and *Methylocystis parvus* were included as outgroup.

We confirmed the taxonomic identification of OTUs through phylogenetic analyses using sequences from *Bathymodiolus* endosymbionts 16S rRNA and other bacteria available in RDP. The resulting tree showed the clear proximity of OTUs to the phylotypes of thiotrophic and the methanotrophic endosymbionts of Lucky Strike’s mussel (Figure 3), thus supporting evidence for the presence of the two chemoautotrophs in mussel gill tissues. This analysis also revealed the proximity of an OTU to *Sulfurovum*. Four of the OTUs were closely related to uncultured bacteria; however, three of the OTUs formed an independent clade from methanotrophic and thiotrophic endosymbionts and one of the OTUs grouped more closely to the methanotrophic endosymbiont (Figure 3). Furthermore, one OTU clustered with *Psychromonas* and two OTUs to uncultured *Spirochaetes* (data not shown). These three latter OTUs were amplified with a different primer set from the OTUs above described and thus were not included in the same alignments for the phylogenetic tree shown.

### 2.5. *B. azoricus* Gill Tissue Microbial Community

The comparison of the taxonomical profiles generated from metabolic transcript binning and 16S ribosomal sequencing revealed discordant results as to which organisms were present in the mussel gill microbial community. This discordance was probably due to the results incurred from different approaches used to obtain taxonomic identification. Transcript binning retrieves the organisms identified by the top BLAST hits, and thus transcripts originating in the same organism can match genes from different organisms if its genome sequence is not yet available. The second approach identifies organisms based on the hypervariable regions of ribosomal DNA by comparison to full 16S ribosomal databases. In accordance with this, if the organisms have not been described yet, this analysis reports information on the closest relative. 

Despite these caveats, the results from the two taxonomic approaches support the presence of a gammaproteobacterial thiotrophic endosymbiont and a close relative of the epsilonbacterium *Sulfurovum*. The transcripts of the thiotrophic endosymbiont most probably correspond to the transcripts affiliated in Candidatus *Ruthia magnifica*, the *Calyptogena* endosymbiont and other bacteria identified through transcript binning. Considering the number of sequences used to generate each OTU in the 16S ribo tag analyses (Sequences column, Table 3) in comparison with the relative proportion between the microorganisms detected, the thiotrophic endosymbiont would be the dominant organism in the gill tissues. Thus, it is expected that most of the transcripts originated in this endosymbiont, assuming that the respective gene expression is proportionally correlated to the number of microorganisms. However, these inferences must be taken with caution since the nature of the starting RNA material for this experiment may potentially introduce a bias in the number of sequencing reads, as it is dependent on the relative gene expression of different community members. 

Interestingly, evidence from this analysis supported the identification of a close relative of *Sulfurovum*, through 16S rRNA pyrosequencing, and the affiliation of expressed genes in epsilonproteobacteria as the third most binned group. Epsilonbacteria from deep sea are chemolithoautotrophs with different metabolisms; some bacteria are hydrogen-oxidizing, thermophilic chemolitoautotrophs, while others are sulfur-oxidizers [50], such as the case of *Sulfurovum* [51]. Additionally, in deep sea hydrothermal vents, epsilonbacteria has also been found in symbiotic relationships, for example, as an endosymbiont of Alviniconcha gastropods from the Central Indian Ridge [52] or as epibionts of *Rimicaris exoculata* at MAR [53,54]. In mussels, there is no description of endosymbionts originating in this bacterial class, and most probably, the transcripts found originated in free living bacteria. However, further investigation is required to clarify the role of epsilonbacteria associations, either with the vent mussel or the vent biological environment. 

## 3. Experimental Section

### 3.1. Animal Collection

Mussels were collected from the hydrothermal vent field Lucky Strike (37°13.52′N, 32°26.18′W; 1700 m depth) on the MAR with the American R/V *Revelle* using the ROV Jason II (Woods Hole Oceanographic Institution), during the MAR08 cruise led by Chief Scientist Dr. Anne-Louise Reysenbach. 

### 3.2. Identification of Transcripts of Bacterial Origin in the Transcriptome of Mussel Gills

In a previous work, we sequenced the transcriptome of a normalized cDNA library from the gill tissues of *B. azoricus* by massive parallel 454 pyrosequencing [17]. Sequencing produced 778,996 raw nucleotide reads, which after removal of low quality terminal regions, removal of SMART adaptors and poly-A masking were assembled with MIRA [55] (version 3.0.5), with default parameters. These contigs translated into 39,425 amino acid sequences, of which 22,023 corresponded to known proteins in the NCBI non-redundant protein database, 15,839 had conserved protein domains, as detected by InterPro functional classification and 9584 matched Gene Ontology terms. We compiled the transcriptome data in a SQL database developed as an information management system, the DeepSeaVent [56]. Upon observation of transcripts matching bacterial phylotypes, we searched the DeepSeaVent for contigs matching at least one bacterial phylotype within the top 20 BLAST hits, thereby identifying 3522 transcripts. The unique identifiers of the contigs (gi accession number) were retrieved and translated into the taxon ID using the information provided by NCBI (National Center for Biotechnology Information) taxonomy. A custom script based on BioPerl module bio::db::taxonomy, version 1.6 [57] linked the taxon of interest to the superkingdom Bacteria to verify whether the BLASTx hit corresponded to canonical Eubacterial sequences [17].

### 3.3. Annotation of Transcripts of Bacterial Origin and Transcript Binning

Transcripts corresponding to bacterial hits were submitted to the open source MetaGenome Rapid Annotation using Subsystem Technology (MG-RAST), version 3.1.2 [18], for assignment of gene function through comparison to non-redundant protein databases: GO, IMG, KEGG, NCBI (RefSeq & GenBank), SEED, UniProt, eggNOG and PATRIC [58]. Annotation parameters were set at a maximum *e*-value of 10^–5^, a minimum of 50% identity cutoff and a minimum alignment cutoff of 50. The taxonomic affiliation of the transcripts derived from the MG-RAST annotation against M5NR, by the lowest common ancestor method, parameterized at a maximum *e*-value of 10^−30^, a minimum of 50% identity cutoff and a minimum alignment cutoff of 50. 

### 3.4. 16S rRNA Amplicon Sequencing of the Gill’s Microbiome

The bacterial diversity in the community was characterized by sequencing of the V6 hypervariable region of the 16S rRNA amplicon library by 454 sequencing. Two micrograms of total RNA, initially used to generate the transcriptome library, were reverse-transcribed with random primers using the ThermoScript™ RT-PCR (Invitrogen, CA, USA) following manufacturer’s instructions. Using the cDNA as template, the V6 region was amplified, using barcoded fusion primers with the Roche-454 A Titanium sequencing adapters, a six-base barcode sequence and forward 5′-ATGCAACGCGAAGAACCT-3′, 5′-AATTGGABTCAACGCC-3′, 5′-GAGGWGGTGCATGGC-3′ and reverse 5′-TAGCGATTCCGACTTCA-3′ primers [59]. Each primer pair generated amplicons of different sizes. Two replicate PCR were amplified from the same sample for each primer set, quantified by fluorimetry with PicoGreen (Invitrogen, CA, USA), pooled at equimolar concentrations and sequenced in the A direction with GS 454 FLX Titanium chemistry, according to manufacturer’s instructions (Roche, 454 Life Sciences, Brandford, CT, USA). Raw pyrosequencing reads were separated according to barcode identifiers and processed through quality filters to remove sequences that did not have a complete forward primer; had less than two undefined nucleotides and were shorter than 100 bp. Additionally, the 3′ ends were trimmed for average quality score ≤15, based on a seven base pair window. After filtering, reads were additionally trimmed for the A and B sequence adaptors and the barcode. The high quality sequences were clustered together by uclust v2.1 [60] with a similarity of 97%. The clustered sequences were then assembled by Cap3 [61] to produce OTUs (Operational Taxonomic Units). The OTUs were searched by NCBI BLAST against RDP (release 10 update 24) with a cutoff of 1e^−50^ to identify the taxa. Chimeras were identified by BLAST, through the confirmation of whether different fragments of the same OTU matched only the same hit. To improve the accuracy of the results, a bootstrap method was included, where OTUs were replicated 100 times and changed in 10% by seqboot application from PHYLIP package [62]. Only sequences with 70% bootstrap support of the same taxonomy were identified. 

### 3.5. Phylogenetic Analysis

OTU sequences were searched by BLAST in the RDP database [46] to retrieve 16S RNA sequences of closely-related microorganisms and aligned with MUSCLE module from MEGA v5 [63]. The alignment, with at least 250 bp, was used to reconstruct the phylogenetic relationship by the Maximum Likelihood method based on the Jukes-Cantor model [64]. One thousand bootstrap replicates were performed to test the support of each node on the trees [65].

### 3.6. Accession Numbers

The entire set of transcriptome reads was submitted to the GenBank Sequence Read Archive under the accession number SRA024338.

## 4. Conclusions

Analysis of *Bathymodiolus azoricus* gills transcriptome revealed transcripts of bacterial origin among mussel transcripts. This discovery suggested that the study of these transcripts could provide an insight into the mussel endosymbionts and probably other bacteria gravitating around the mussel. The transcripts were annotated for function and binned for taxonomical affiliation, thus characterizing the microbial community of *B. azoricus* from the Lucky Strike vent field.

The goals of this study were to identify the organisms present in the gill tissue and infer their functions. Indeed, we gathered evidence for the presence of the thiotrophic and the methanotrophic endosymbionts as expected, but also evidence for the occurrence of a microorganism closely related to *Sufurovum*, a free living epsilon proteobacterium. On the other hand, the functional analysis revealed enzymes involved in sulfur and methane oxidation as would be expected, but these were not sufficient to characterize completely the molecular pathways underlying sulfur and methane oxidation within the host. Nevertheless, this study demonstrated how metatranscriptomics is a powerful approach for analyzing complex bacterial communities and for capturing gene expression patterns in microbial associations, such as deep sea host-symbiont interactions, even in small datasets as the one analyzed in this report, contributing to the understanding of microbial communities in natural environments.

## Supplementary Files

Supplementary File 1: PDF-Document (PDF, 407 KB)
