# Supplementary material for: The Transcriptome of Bathymodiolus azoricus Gill Reveals Expression of Genes from Endosymbionts and Free-Living Deep-Sea Bacteria
Source: Mar Drugs. 2012 Aug 20;10(8):1765–83. doi: 10.3390/md10081765 (PMC3447338; doi:10.3390/md10081765)
Supplement: Supplementary File 1: — PDF-Document (PDF, 407 KB) [file marinedrugs-10-01765-s001.pdf]

Supplementary Information

Table S1. Distribution of transcripts annotated according to the SEED subsystems in MG-RAST. Transcript gene function was assigned in MG-RAST, with the following parameters: maximum e-value of 10^{-5}, minimum of 50% identity cutoff and minimum alignment cutoff of 50.

Subsystem Hierarchy 1	Subsystem Hierarchy 2	Subsystem Name	# transcripts
Amino Acids and Derivatives	Alanine, serine, and glycine	Alanine biosynthesis	1
		Glycine Biosynthesis	1
		Glycine cleavage system	2
		Serine Biosynthesis	1
	Arginine; urea cycle, polyamines	Arginine Biosynthesis	8
		Polyamine Metabolism	2
		Urea decomposition	1
	Aromatic amino acids and derivatives	Aromatic amino acid degradation	1
		Chorismate: Intermediate for synthesis of PAPA antibiotics, PABA, anthranilate, 3-hydroxyanthranilate and more	2
		Chorismate Synthesis	6
		Common Pathway For Synthesis of Aromatic Compounds (DAHP synthase to chorismate)	13
		Phenylalanine synthesis	2
		Tryptophan synthesis	5
	Branched-chain amino acids	Branched-Chain Amino Acid Biosynthesis	10
		HMG CoA Synthesis	1
		Valine degradation	1
	Histidine Metabolism	Histidine Biosynthesis	6
	Lysine, threonine, methionine, and cysteine	Cysteine Biosynthesis	5
		Lysine Biosynthesis DAP Pathway	3
		Methionine Biosynthesis	8
		Methionine Degradation	4
		Threonine and Homoserine Biosynthesis	9
		Threonine degradation	1
	Osmotic stress	Choline and Betaine Uptake and Betaine Biosynthesis	1
	Proline and 4-hydroxyproline	Proline, 4-hydroxyproline uptake and utilization	1

Table S1. *Cont.*

Carbohydrates	Aminosugars	Chitin and N-acetylglucosamine utilization	15
	CO2 fixation	CO2 uptake, carboxysome	8
		Calvin-Benson cycle	7
	Carbohydrates	beta-glucuronide utilization	1
	Central carbohydrate metabolism	Dehydrogenase complexes	6
		Dihydroxyacetone kinases	1
		Entner-Doudoroff Pathway	6
		Glycolate, glyoxylate interconversions	5
		Methylglyoxal Metabolism	1
		Pentose phosphate pathway	7
		Pyruvate:ferredoxin oxidoreductase	7
		Pyruvate Alanine Serine Interconversions	1
		Pyruvate metabolism I: anaplerotic reactions, PEP	2
		Pyruvate metabolism II: acetyl-CoA, acetogenesis from pyruvate	6
		TCA Cycle	1
	Clustering-based subsystems	Cluster Ytf and putative sugar transporter	1
		Putative sugar ABC transporter (ytf cluster)	1
	Di- and oligosaccharides	Lactose and Galactose Uptake and Utilization	6
		Maltose and Maltodextrin Utilization	3
		Sucrose Metabolism	3
	Fermentation	Acetoin, butanediol metabolism	1
		Acetyl-CoA fermentation to Butyrate	1
		Fermentations: Mixed acid	1
	Monosaccharides	D-gluconate and ketogluconates metabolism	3
		D-ribose utilization	1
		Deoxyribose and Deoxynucleoside Catabolism	9
		L-Arabinose utilization	1
		L-fucose utilization temp	6
		Mannose Metabolism	1
		Xylose utilization	2
	One-carbon Metabolism	Methanogenesis	2
		Serine-glyoxylate cycle	7
	Organic acids	Glycerate metabolism	1
		Glyoxylate Synthesis	2
	Sugar alcohols	Glycerol and Glycerol-3-phosphate Uptake and Utilization	1
		Hexitol degradation	1

Table S1. *Cont.*

Cell Division and Cell Cycle	Cell cycle in Prokaryota	Bacterial Cell Division	9
		Bacterial Cytoskeleton	19
		Two cell division clusters relating to chromosome partitioning	3
Cell Wall and Capsule	Capsular and extracellular polysacchrides	CMP-N-acetylneuraminate Biosynthesis	1
		Colanic acid biosynthesis	1
		O-Methyl Phosphoramidate Capsule Modification in Campylobacter	1
		Rhamnose containing glycans	3
		Sialic Acid Metabolism	1
		dTDP-rhamnose synthesis	2
	Cell Wall and Capsule	Peptidoglycan Biosynthesis	11
		UDP-N-acetylmuramate from Fructose-6-phosphate Biosynthesis	2
		YjeE	3
	Gram-Negative cell wall components	KDO2-Lipid A biosynthesis	8
		LOS core oligosaccharide biosynthesis	4
		Lipid A-Ara4N pathway (Polymyxin resistance)	3
		Lipopolysaccharide assembly	5
		Teichoic and lipoteichoic acids biosynthesis	3
		Teichuronic acid biosynthesis	1
Clustering-based subsystems	Biosynthesis of galactoglycans and related lipopolysacharides	CBSS-258594.1.peg.3339	2
	CRISPRs and associated hypotheticals	CBSS-216592.1.peg.3534	1
	Carotenoid biosynthesis	CBSS-320388.3.peg.3759	1
	Cell Division	CBSS-281309.1.peg.50	5
	Clustering-based subsystems	CBSS-160492.1.peg.550	2
		CBSS-176279.3.peg.868	1
		CBSS-176280.1.peg.1561	4
		CBSS-176299.4.peg.1996	4
		CBSS-224911.1.peg.435	1
		CBSS-228410.1.peg.134	1
		CBSS-235.1.peg.567	3
		CBSS-243277.1.peg.4359	3
		CBSS-251221.1.peg.1863	2

Table S1. *Cont.*

		CBSS-257314.1.peg.752	4
		CBSS-261591.3.peg.3916	5
		CBSS-281090.3.peg.464	2
		CBSS-288681.3.peg.1039	2
		CBSS-290633.1.peg.1906	7
		CBSS-296591.1.peg.2330	3
		CBSS-312309.3.peg.1965	2
		CBSS-316057.3.peg.1294	24
		CBSS-316057.3.peg.563	1
		CBSS-316279.3.peg.746	1
		CBSS-320372.3.peg.6046	2
		CBSS-335283.3.peg.454	19
		CBSS-342610.3.peg.1794	6
Clustering-based subsystem (continued)	Clustering-based subsystems (continued)	CBSS-345072.3.peg.1318	2
		CBSS-56780.10.peg.1536	1
		CBSS-630.2.peg.3360	1
		Conserved gene cluster associated with Met-tRNA formyltransferase	4
		Conserved gene cluster possibly involved in RNA metabolism	2
		EC49-61	2
		EC699-706	1
		Putative hemin transporter	1
	Cytochrome biogenesis	CBSS-196164.1.peg.461	1
	DNA polymerase III epsilon cluster	CBSS-342610.3.peg.1536	14
	Fatty acid metabolic cluster	CBSS-218491.3.peg.427	9
		CBSS-246196.1.peg.364	4
	Hypothetical associated with RecF	Hypothetical Coupled to RecF	1
	Hypothetical in Lysine biosynthetic cluster	CBSS-323850.3.peg.3269	1
	Hypothetical lipase related to Phosphatidate metabolism	CBSS-316407.3.peg.1371	3
	Hypothetical protein possible functionally linked with Alanyl-tRNA synthetase	CBSS-257314.1.peg.488	5

Table S1. *Cont.*

	Isoprenoid/cell wall biosynthesis: PREDICTED UNDECAPRENYL DIPHOSPHATE PHOSPHATASE	CBSS-83331.1.peg.3039	7
	Lipoprotein B cluster	CBSS-221988.1.peg.2137	1
	PmbA Related Hypothetical	PmbA and a Hypothetical Protein	1
	Probably GTP or GMP signaling related	CBSS-176299.4.peg.1292	4
	Probably Pyrimidine biosynthesis-related	CBSS-306254.1.peg.1508	1
	Probably Ybbk-related hypothetical membrane proteins	CBSS-316057.3.peg.659	1
	Probably organic hydroperoxide resistance related hypothetical protein	CBSS-269482.1.peg.1294	3
	Protein export?	CBSS-393121.3.peg.2760	12
	Putative GGDEF domain protein related to agglutinin secretion	CBSS-323850.3.peg.3284	3
	Putative asociate of RNA polymerase sigma-54 factor rpoN	CBSS-316057.3.peg.1308	3
	Putrescine/GABA utilization cluster-temporal,to add to SSs	GABA and putrescine metabolism from cluters	2
	Recombination related cluster	CBSS-258594.1.peg.613	12
	Ribosomal Protein L28P relates to a set of uncharacterized proteins	A Gram-positive cluster that relates ribosomal protein L28P to a set of uncharacterized proteins	1
	Ribosome-related cluster	A Gammaproteobacteria Cluster Relating to Translation	1
		CBSS-138119.3.peg.2719	2
	TldD cluster	CBSS-354.1.peg.2917	2
	Translation	CBSS-326442.4.peg.1852	7
	Urate degradation	CBSS-205922.3.peg.1809	3
	tRNA sulfuration	CBSS-89187.3.peg.2957	2

Table S1. *Cont.*

Cofactors, Vitamins, Prosthetic Groups, Pigments	Biotin	Biotin biosynthesis	2
	Coenzyme A	Coenzyme A Biosynthesis	3
	Folate and pterines	5-FCL-like protein	2
		Folate Biosynthesis	16
		Molybdopterin biosynthesis	4
		Pterin metabolism 3	3
		YgfZ	4
	Isoprenoids	Isoprenoid Biosynthesis	7
	Lipoic acid	Lipoic acid metabolism	3
	NAD and NADP	NAD and NADP cofactor biosynthesis global	3
	Pyridoxine	Pyridoxin (Vitamin B6) Biosynthesis	2
	Quinone cofactors	Menaquinone and Phylloquinone Biosynthesis	1
		Pyrroloquinoline Quinone biosynthesis	1
	Tetrapyrroles	Chlorophyll Degradation	1
		Cobalamin synthesis	1
		Coenzyme B12 biosynthesis	2
		Heme and Siroheme Biosynthesis	8
	Thiamine and thiamine pyrophosphate	Thiamin biosynthesis	2
DNA Metabolism	DNA Metabolism	DNA structural proteins, bacterial	1
		Restriction-Modification System	13
		YcfH	1
	DNA repair	DNA Repair Base Excision	3
		DNA repair, UvrABC system	4
		DNA repair, bacterial	1
		DNA repair, bacterial RecFOR pathway	2
		Rad50-Mre11::SbcD-SbcC DNA Repair Module	1
	DNA replication	DNA-replication	15
		DNA topoisomerases, Type II, ATP-dependent	2
Fatty Acids and Lipids	Fatty Acids and Lipids	Polyhydroxybutyrate metabolism	1
	Fatty acids	Fatty Acid Biosynthesis FASII	2
	Phospholipids	Glycerolipid and Glycerophospholipid Metabolism in Bacteria	3
Macromolecular Synthesis	Control of Macromolecuar Synthesis	CBSS-243277.1.peg.511	1

Table S1. *Cont.*

Membrane Transport	ABC transporters	ABC transporter tungstate (TC 3.A.1.6.2)	1
	Lipoprotein sorting	Lipoprotein sorting system	2
	Membrane Transport	Transport of Zinc	2
	Uni- Sym- and Antiporters	Na(+) H(+) antiporter	6
Metabolism of Aromatic Compounds	Metabolism of central aromatic intermediates	Catechol branch of beta-ketoadipate pathway	1
		Phenylacetate pathway of aromatic compound degradation	1
	Peripheral pathways for catabolism of aromatic compounds	Benzoate degradation	1
		Biphenyl Degradation	1
Miscellaneous	Miscellaneous	Transporters In Models	6
Motility and Chemotaxis	Flagellar motility in Prokaryota	Flagellum	6
	Social motility and nonflagellar swimming in bacteria	Bacterial motility:Gliding	3
Nitrogen Metabolism	Nitrogen Metabolism	Ammonia assimilation	1
		Denitrification	1
		Nitrate and nitrite ammonification	5
Nucleosides and Nucleotides	Detoxification	Nudix proteins (nucleoside triphosphate hydrolases)	3
	Nucleosides and Nucleotides	De Novo Pyrimidine Synthesis	6
		Hydantoin metabolism	1
		Purine Utilization	3
		Ribonucleotide reduction	7
	Purines	De Novo Purine Biosynthesis	5
		Purine conversions	5
	Pyrimidines	Pyrimidine utilization	2
		pyrimidine conversions	3
Phosphorus Metabolism	Phosphorus Metabolism	Alkylphosphonate utilization	1
		Phosphate metabolism	8
		Phosphonate metabolism	3
Potassium metabolism	Potassium metabolism	Glutathione-regulated potassium-efflux system and associated functions	7

Table S1. *Cont.*

Protein Metabolism	Protein biosynthesis	Asp-tRNA(Asn) transamidation	4
		Ribosome LSU bacterial	29
		Ribosome LSU mitochondrial	1
		Ribosome SSU bacterial	10
		Ribosome activity modulation	1
		Translation factors bacterial	1
		Universal GTPases	11
		tRNA aminoacylation	27
		Proteasome bacterial	5
		Proteasome eukaryotic	11
		Protein degradation	2
		Proteolysis in bacteria, ATP-dependent	1
	Protein folding	GroEL GroES	1
		Peptidyl-prolyl cis-trans isomerase	2
		Periplasmic disulfide interchange	1
		Protein chaperones	14
	Protein processing and modification	Inteins	1
	Secretion	General Secretion Pathway	10
		General secretory pathway (Sec-SRP) complex (TC 3.A.5.1.1)	8
		Twin-arginine translocation system	1
Regulation and Cell signaling	Programmed Cell Death and Toxin-antitoxin Systems	Toxin-antitoxin systems (other than RelBE and MazEF)	1
	Proteolytic pathway	Coagulation cascade	5
	Regulation and Cell signaling	Glutaredoxins	4
		MazEF toxin-antitoxing (programmed cell death) system	2
		Phytochromes	1
		Two-component regulatory systems in Campylobacter	2
		cAMP signaling in bacteria	8

Table S1. *Cont.*

Respiration	ATP synthases	F0F1-type ATP synthase	11
	Electron accepting reactions	Anaerobic respiratory reductases	4
		Terminal cytochrome C oxidases	7
		Na(+)-translocating NADH-quinone oxidoreductase and rnf-like group of electron transport complexes	8
		NiFe hydrogenase maturation	1
		Respiratory Complex I	11
		Respiratory dehydrogenases 1	4
		Succinate dehydrogenase	1
	Respiration	Biogenesis of c-type cytochromes	2
		Biogenesis of cbb3-type cytochrome c oxidases	2
		Biogenesis of cytochrome c oxidases	2
		Formate hydrogenase	3
		Soluble cytochromes and functionally related electron carriers	1
	Sodium Ion-Coupled Energetics	Na+ translocating decarboxylases and related biotin-dependent enzymes	1
RNA Metabolism	RNA Metabolism	Group II intron-associated genes	36
	RNA processing and modification	ATP-dependent RNA helicases, bacterial	1
		Polyadenylation bacterial	3
		Queuosine-Archaeosine Biosynthesis	4
		RNA processing and degradation, bacterial	5
		eukaryotic rRNA modification and related functions	1
		rRNA modification Bacteria	1
		tRNA modification E.coli	14
		tRNA processing	3
	Transcription	RNA polymerase II	1
		RNA polymerase bacterial	7
		Transcription factors bacterial	5
Secondary Metabolism	Plant hormones	Auxin biosynthesis	2
	Biologically active compounds in metazoan cell defence and differentiation	Quinolinic acid and its derivatives	2

Table S1. *Cont.*

Stress Response	Cold shock	Cold shock, CspA family of proteins	1
	Osmotic stress	Hyperosmotic ectoine biosynthesis	1
	Oxidative stress	Glutathione Redox Metabolism	1
		Oxidative stress	8
		Protection from Reactive Oxygen Species	1
	Stress Response	Acid resistance mechanisms	1
		Bacterial hemoglobins	2
		Carbon Starvation	2
		Copper homeostasis	10
		Dimethylarginine metabolism	1
		Flavohaemoglobin	5
		Heat shock dnaK gene cluster extended	2
Sulfur Metabolism	Inorganic sulfur assimilation	Sulfate assimilation	1
	Organic sulfur assimilation	Alkanesulfonate assimilation	1
		Utilization of glutathione as a sulphur source	1
	Sulfur Metabolism	Galactosylceramide and Sulfatide metabolism	3
		Sulfate reduction-associated complexes	2
		Sulfur oxidation	12
Unclassified	Unclassified	Branched chain amino acid degradation regulons	2
		CBSS-224911.1.peg.7673	3
		CBSS-393121.3.peg.1913	2
		CBSS-412883.3.peg.2513	1
		CBSS-83332.1.peg.3803	5
		CBSS-87626.3.peg.3639	1
		CR clusters Euk 7 GlucoseP isomerase	1
		Experimental-Ubiquinone BiosynthesisVDC	3
		Iron-sulfur cluster assembly	9
		Iron-sulfur experimental	9
		RuvABC plus a hypothetical	3
		Selenoprotein O	1
		Sugar catabolome in Shewanella species	2
		YgfZ-Fe-S	1
		YgfZ-Fe-S clustering	1
		Zinc regulated enzymes	1

Table S1. *Cont.*

Virulence, Disease and Defense	Adhesion	Adhesins in Staphylococcus	1
	Invasion and intracellular resistance	Listeria surface proteins: Internalin-like proteins	2
	Iron Scavenging Mechanisms	Hemin transport system	2
		Pyoverdine biosynthesis new	1
		Transport of Iron	1
	Pathogenicity islands	Staphylococcal pathogenicity islands SaPI	1
	Posttranslational modification	N-linked Glycosylation in Bacteria	6
	Resistance to antibiotics and toxic compounds	Acriflavin resistance cluster	7
		Beta-lactamase	2
		Cobalt-zinc-cadmium resistance	8
		Methicillin resistance in Staphylococci	16
		Multidrug Resistance Efflux Pumps	1
		Resistance to fluoroquinolones	5
	Type III, Type IV, ESAT secretion systems	ESAT-6 proteins secretion system in Firmicutes	1
		Mannose-sensitive hemagglutinin type 4 pilus	1
		Type 4 secretion and conjugative transfer	20
		Type III secretion system orphans	1
		Type IV pilus	6
	Virulence	C jejuni colonization of chick caeca	1
		Ton and Tol transport systems	5

Table S2. Distribution of transcripts annotated according to the COG in MG-RAST. Transcript gene function was assigned in MG-RAST, with the following parameters: maximum e-value of 10^{-5}, minimum of 50% identity cutoff and minimum alignment cutoff of 50.

Major Categories	Description	# Hits
CELLULAR PROCESSES AND SIGNALING	Cell cycle control, cell division, chromosome partitioning	4
	Cell wall/membrane/envelope biogenesis	25
	Defense mechanisms	4
	Intracellular trafficking, secretion, and vesicular transport	12
	Posttranslational modification, protein turnover, chaperones	27
	Signal transduction mechanisms	10
INFORMATION STORAGE AND PROCESSING	RNA processing and modification	1
	Replication, recombination and repair	30
	Transcription	11
	Translation, ribosomal structure and biogenesis	52
METABOLISM	Amino acid transport and metabolism	42
	Carbohydrate transport and metabolism	21
	Coenzyme transport and metabolism	28
	Energy production and conversion	45
	Inorganic ion transport and metabolism	18
	Lipid transport and metabolism	14
	Nucleotide transport and metabolism	20
	Secondary metabolites biosynthesis, transport and catabolism	2
POORLY CHARACTERIZED	Function unknown	6
	General function prediction only	44
